# TGFBI Production by Macrophages Contributes to an Immunosuppressive Microenvironment in Ovarian Cancer

**DOI:** 10.1158/0008-5472.CAN-21-0536

**Published:** 2021-09-24

**Authors:** Laura S.M. Lecker, Chiara Berlato, Eleni Maniati, Robin Delaine-Smith, Oliver M.T. Pearce, Owen Heath, Samuel J. Nichols, Caterina Trevisan, Marian Novak, Jacqueline McDermott, James D. Brenton, Pedro R. Cutillas, Vinothini Rajeeve, Ana Hennino, Ronny Drapkin, Daniela Loessner, Frances R. Balkwill

**Affiliations:** 1 Barts Cancer Institute, London, United Kingdom.; 2 Fondazione Istituto di Ricerca Pediatrica Città della Speranza, Padova, Italy.; 3 Department of Women and Children Health, University of Padova, Padova, Italy.; 4 Department of Medical Oncology, Dana-Farber Cancer Institute and Harvard Medical School, Boston, Massachusetts.; 5 Cancer Research UK Cambridge Institute, University of Cambridge, Cambridge, United Kingdom.; 6 Cancer Research Center of Lyon, UMR INSERM 1052, Lyon, France.; 7 Ovarian Cancer Research Center, Perelman School of Medicine, Philadelphia, Pennsylvania.

## Abstract

The tumor microenvironment evolves during malignant progression, with major changes in nonmalignant cells, cytokine networks, and the extracellular matrix (ECM). In this study, we aimed to understand how the ECM changes during neoplastic transformation of serous tubal intraepithelial carcinoma lesions (STIC) into high-grade serous ovarian cancers (HGSOC). Analysis of the mechanical properties of human fallopian tubes (FT) and ovaries revealed that normal FT and fimbria had a lower tissue modulus, a measure of stiffness, than normal or diseased ovaries. Proteomic analysis of the matrisome fraction between FT, fimbria, and ovaries showed significant differences in the ECM protein TGF beta induced (TGFBI, also known as βig-h3). STIC lesions in the fimbria expressed high levels of TGFBI, which was predominantly produced by CD163-positive macrophages proximal to STIC epithelial cells. *In vitro* stimulation of macrophages with TGFβ and IL4 induced secretion of TGFBI, whereas IFNγ/LPS downregulated macrophage TGFBI expression. Immortalized FT secretory epithelial cells carrying clinically relevant TP53 mutations stimulated macrophages to secrete TGFBI and upregulated integrin αvβ3, a putative TGFBI receptor. Transcriptomic HGSOC datasets showed a significant correlation between TGFBI expression and alternatively activated macrophage signatures. Fibroblasts in HGSOC metastases expressed TGFBI and stimulated macrophage TGFBI production *in vitro*. Treatment of orthotopic mouse HGSOC tumors with an anti-TGFBI antibody reduced peritoneal tumor size, increased tumor monocytes, and activated β3-expressing unconventional T cells. In conclusion, TGFBI may favor an immunosuppressive microenvironment in STICs that persists in advanced HGSOC. Furthermore, TGFBI may be an effector of the tumor-promoting actions of TGFβ and a potential therapeutic target.

**Significance:**

Analysis of ECM changes during neoplastic transformation reveals a role for TGFBI secreted by macrophages in immunosuppression in early ovarian cancer.

## Introduction

There are increasingly compelling data to show that fallopian tube secretory epithelial (FTSE) cells are the progenitors of many high-grade serous ovarian carcinomas (HGSOC; refs. 1–8). The earliest precursors are “p53 signatures,” which further transform into serous tubal intraepithelial carcinomas (STIC) and are found in the fallopian tube (FT) fimbria (FB) of women with and without familial ovarian cancer. These STICs share common features with HGSOC cells (9).

Dysregulation of extracellular matrix (ECM) components is one of the key pathologic processes in neoplastic transformation, where ECM remodeling leads to abnormal cellular behaviour and growth (10, 11). Stromal desmoplasia evoked by malignant cells promotes profound structural changes and tissue stiffening (11–13). Tumor matrix is primarily laid down by fibroblasts but immune cells, in particular macrophages, are also associated with physiologic and abnormal ECM expression (14–16).

Our group has published a proteomic analysis of the matrisome of omental metastasis of HGSOC, revealing a matrix signature that is predictive of patient survival (14). Furthermore, we found that omental metastases of HGSOC were up to 100 times stiffer than healthy omentum (14). However, the ECM of precursor FT lesions has not been widely studied. Whereas metastasizing HGSOC cells attach and infiltrate omental tissue that has relatively low endogenous ECM levels, STIC lesions and early HGSOCs in the ovary are developing in a stroma-rich microenvironment.

It is not clear why and how transformed FTSE cells spread from the fimbrial end of the FT and adhere to the ovarian surface. As the ECM mediates adhesion, migration, and invasion, we propose that understanding the biomechanical and matrisome profiles of the FT and ovary will aid our understanding of early HGSOC.

Here we show that the ECM protein TGF beta induced (TGFBI–also known as βig-h3), is significantly upregulated in STIC lesions and HGSOC stroma in ovaries. We found that tumor-associated macrophages (TAM) were the predominant cell type secreting TGFBI in STIC lesions. Further *in vitro*, *in silico* and mouse model experiments using our recently developed orthotopic HGSOC models (17) led us to conclude that TGFBI is an important component of tumor microenvironments at different sites and stages of HGSOC and that it may contribute to immunosuppression and disease progression.

## Materials and Methods

### Tissue collection

Human ovarian (OV), FT, fimbrial, and HGSOC tissue was obtained from patients at Barts Health NHS Trust, following full written informed consent. Tissues surplus to diagnostic requirements was collected under the Barts Gynae Tissue Bank HTA license number 12199 (REC no: 10/H0304/14 and 15/EE/0151). Study was approved by the East of England Cambridge UK review board and conducted in accordance with the Declaration of Helsinki and the International Ethical Guidelines for Biomedical Research Involving Human Subjects.

### Mechanical characterization

Prior to mechanical indentation measurements, frozen, unfixed FT, FB, and ovary tissue were fully thawed at room temperature in PBS (catalog no. D8537, Sigma-Aldrich) for 30 minutes. Mechanical indentation of the specimens was performed using an Instron ElectroPuls E1000 (Instron) equipped with a 10N load cell (resolution = 0.1 mN) and a flat, rigid cylindrical indenter with a 1 mm diameter (∅I; previously published in ref. 18).

Tissue moduli comparative to those obtained from compression tests were calculated from the obtained load-displacement experimental data with the aid of a corrected mathematical model described in ref. 18.

### Matrisome analysis

Tissue preparation and ECM protein enrichment were performed following Naba and colleagues (19) using the CNMCS (Cytosol/Nucleus/Membrane/Cytoskeleton) Compartmental Protein Extraction kit (pke13011, Cytomol). Samples were run on a linear trap quadrupole Q-Exactive mass spectrometer (Thermo Fisher Scientific) coupled online to nanoflow ultrahigh-pressure liquid chromatography (NanoAcquity, Waters). Peptides were identified by Mascot searches against the SwissProt human protein database. PESCAL (20) was used to obtain peak areas in extracted ion chromatograms of identified peptides and protein abundance determined by the ratio of the sum of peptide areas of a given protein to the sum of all peptide areas. Differential protein abundance was examined using Mann–Whitney *U* test. Mass spectrometry proteomics data are deposited to the ProteomeXchange Consortium via the PRIDE (21) partner repository with dataset identifiers PXD023912 and 10.6019/PXD023912.

### IHC

Samples were fixed in 10% formalin (HT501128, Sigma-Aldrich) and paraffin embedded. Sections were dewaxed, dehydrated, and incubated in antigen unmasking solution (H-3300, Vector Laboratories) in a preheated pressure cooker for 20 minutes. Sections were incubated in 0.6% H_2_O_2_ in methanol for 20 minutes. Staining was performed using the SuperSensitive polymer-HRP kit (QD430-XAKE, Biogenex) according to manufacturer's protocol. Primary antibody [AlphaV 1:200 HPA004856, RRID:AB_1846316, Beta3 1:500 HPA027852, RRID:AB_10601760, POSTN 1:250 HPA012306, RRID:AB_1854827, TGFBI 1:750 HPA017019, RRID:AB_2669511 (Sigma), p53 1:100 IS616 Dako] was diluted (ZUC025, Zytomed Systems) and incubated 1 hour at room temperature. Sections were washed, incubated with Biogenex SuperEnhancer for 20 minutes, washed again, incubated with Biogenex ss label poly-HRP for 30 minutes. Sections were washed before addition of DAB chromogen and counterstaining with hematoxylin (C.I.75290, Merck). Sections were dehydrated, mounted in DPX (06522, Sigma-Aldrich), and examined with Panoramic digital slide scanner (3DHISTECH).

### ISH

We used Advanced Cell Diagnostic kits for probes and protocols. Tissue sections were stained with the TGFBI probe (RNAscope probe Hs-TGFBI, 478491) using the RNAscope 2.5 HD reagent kit-Brown (322300). Slides were scanned using the Panoramic digital slide scanner (3DHISTECH). For dual stains the RNAscope 2.5 HD Duplex Reagent Kit (322430) was used. The TGFBI probe was mixed with one of the following probes diluted 1:50: Hs-CD3-pool-C2 (426621-C2), Hs-CD68-C2 (560591-C2), Hs-CD163-C2 (417061-C2), Hs-MRC1-No-XMm-C3 (564211-C3), Hs-ACTA2-O1-C2 (444771-C2).

### International Cancer Genome Consortium and The Cancer Genome Atlas analysis

The ICGC_OV read counts across 93 samples were extracted from the exp_seq.OV-AU.tsv.gz file in the ICGC data repository Release 20 (http://dcc.icgc.org). Genes that achieved at least one read count in at least 10 samples were selected, producing 18,010 filtered genes. Variance stabilizing transformation was applied using the rlog function. For TCGA_OV set, the normalized gene expression data profiled by Affymetrix U133a 2.0 Array version 2015-02-24 were downloaded from UCSC Cancer Browser. Correlation of TGFBI and CD163 used Pearson correlation. For CIBERSORT analysis, The Cancer Genome Atlas (TCGA) and International Cancer Genome Consortium (ICGC) datasets were ordered by TGFBI expression to identify the top and lower 30% of the samples (TGFBI high and TGFBI low). These data were extracted (*N* = 200 samples/group for TCGA and *N* = 30 samples/group for ICGC) and used as input in CIBERSORT, RRID:SCR_016955, (https://cibersort.stanford.edu/) run with the LM22 leukocyte signature matrix (22).

### Macrophage differentiation

Human peripheral blood mononuclear cells from anonymous healthy donors were obtained from NHS Blood and Transplant service. PBMCs were isolated using Ficoll-Paque PLUS (17-1440-03 AG, GE Healthcare). Monocytes were isolated from PBMCs by CD14 microbeads (130-050-201, Miltenyi Biotec) and magnetic isolation on LS columns (130-042-401, Miltenyi Biotec). Monocytes were treated for 7 days with 100 ng/mL MCSF (574806, BioLegend) and polarized with IL4 20 ng/mL (200-04), IL10 20 ng/mL (200-10), IL13 20 ng/mL (200-13), TGFβ 20 ng/mL (100-21C), IFNγ 10 ng/mL (300-02), from Peprotech and LPS 100 ng/mL L2630 Sigma-Aldrich in 100 ng/mL MCSF for 72 hours. TGFβ inhibitor, SB431542 (S4317, Sigma) was used at 10 μmol/L.

### Cell lines and culture conditions

FNE01 and FNE02 were received from University of Miami (Miami, FL) and cultured on Primaria flasks with the FOMI Medium as described previously (23). The other FTSE cell lines and p53 mutant lines were grown in serum-free WIT-P medium (CM-0101, Cellaria) without antibiotics with 100 ng/mL cholera toxin (C8052, Sigma-Aldrich) onto human placental collagen IV (C7521, Sigma-Aldrich) coated plates as described previously (24) and passaged with 0.05% trypsin/EDTA (15400-054, Gibco). HGSOC cell line G164 (25) was grown in DMEM/F12/Glutamax (31331-093, Life Technologies) 10% FCS, 100 μg/mL pen/strep (15140-122, Gibco). FT318 p53 mutant were authenticated by short tandem repeat (STR) sequencing with LCG at the beginning of the project. G164 cells were authenticated by STR sequencing with ATCC (135-XV) at the end of the project. Mouse cell line HGS2 (17) was grown in DMEM/F12 with Glutamax with 4% FBS, 100 μg/mL pen/strep (15140-122, Gibco) and ITS (51300-044, Gibco), murine EGF (E4127, Sigma), hydrocortisone (H0135, Sigma), and anti-anti (15240-062, Gibco). Routine testing for *Mycoplasma* contamination using the MycoAlert PLUS *Mycoplasma* Detection Kit (catalog no. LT07-710, Lonza) has been consistently negative. Cell lines were used within six passages from thawing.

### Transwell coculture assays

Differentiated macrophages (1.5 × 10^6^/well) were cultured in 6-well plates and human fibroblasts isolated from omentum (0.35 × 10^6^/well) seeded in a 6-well transwell dish pore size of 0.4 μm (10380291, Thermo Fisher Scientific) overlaying the macrophages for 3 days. Macrophages were cultured in 2 mL RPMI1640 (31870-074, Invitrogen) with 10% FCS (SV30160.03, HyClone), 100 μg/mL pen/strep, and 100 ng/mL rhMCSF (574806, BioLegend). Fibroblasts were cultured in 2 mL DMEM/F12/Glutamax (31331-093, Life Technologies), 10% FCS, 100 μg/mL pen/strep.

### Omental fibroblasts isolation

Omental samples were diced with scalpels, then digested in 20ml of 0.5 mg/mL collagenase (17018029, Thermo Fisher Scientific) in DMEM (41966-029, Gibco) with 5% FCS at 37°C under agitation (55 rpm) for 75 minutes. Dissociated tissue was disaggregated with a Pasteur pipette and filtered through 250 μm tissue strainers (87791, Thermo Fisher Scientific). Filtered cells were collected and spun for 5 minutes at 200× *g* at room temperature. The pellet was cultured in DMEM/F12/Glutamax (31331-093, Life Technologies) with 10% FCS and 100 μg/mL pen/strep and frozen.

### Flow cytometry

Cells were trypsinized, washed in 2% BSA FACS buffer and stained in primary antibody or directly conjugated antibodies at 5 μg/mL at 4°C for 45 minutes as specified in the Antibodies list. Cells were washed twice with 0.5% BSA FACS buffer and incubated in secondary antibody (1:250 Anti-mouse AlexaFluor 568, A10037, RRID:AB_2534013, Life Technologies) in 0.5% BSA FACS buffer with fixable viability dye (FVD450nm; 65-0863-14, eBioscience) at 1:250. After 30 minutes at 4°C and three washing steps with 2% BSA FACS buffer, cells were fixed with 2% formalin (HT501128, Sigma-Aldrich) in 2% BSA FACS buffer for 10 minutes and washed. Mouse omenta were digested in HBSS (9374543, Gibco) supplemented with collagenase (C9263, Sigma) and DNAase I for 20 minutes at 37°C and filtered through a 70 μm strainer. Cells were washed and resuspended in FACS buffer (PBS + 2% FBS + 2 mmol/L EDTA). The cell suspension was stained in FACS buffer for 30 minutes at 4°C with the antibodies listed in Supplementary Table S1. Cells were washed and stained with FVD506 (65-0866-14, eBioscience) for 25 minutes at 4°C. After fixation, cells were analyzed on a LSR Fortessa II (BD Biosciences) and results analysed with FlowJo v10.2, RRID:SCR_008520, (Treestar Inc.).

### Real-time PCR

RNA was isolated with RNeasy Microkit (74004, Quiagen) according to manufacturer's instructions with additional on-column DNase digestion. Up to 2 μg of RNA was reverse transcribed using the High-capacity cDNA RT kit (4368814, Thermo Fisher Scientific). A total of 5 μg of cDNA in a volume of 9 μL was used in combination with 10 μL iTaq Universal probe supermix (172-5132, Bio-Rad) and 1 μL of the respective primer probe: TGFBI Hs00932747_m1 3-11 63, GAPDH HS02758991_g1 3-8 93 (Thermo Fisher Scientific). Samples were run in triplicates using a StepOnePlus real-time PCR machine (Applied Biosystems). ΔC_t_ values were calculated by subtracting *C*_t_ of the housekeeping gene from each *C*_t_ value of the gene of interest. ΔΔ*C*_t_ was used to compare fold change expression between groups.

### Western blotting

Cells were lysed with RIPA buffer (R0278, Sigma) containing 1:10 complete mini-EDTA protease inhibitor (11836153001, Roche) and 1:100 phosphatase inhibitors (P5726, Sigma). Protein concentration was determined using BCA assay. 20–35 μg samples were loaded on 4%–12% NuPAGE Bis-Tris gels (NP0335BOX, NP0336BOX, Invitrogen). Samples were run in 1× MOPS SDS running buffer (NP0001, Invitrogen) with NuPAGE anti-oxidant solution (NP0005, Invitrogen), and transferred onto a membrane (NEF1002001PK, Perkin Elmer) in 1× NuPAGE transfer buffer (NP0006-1, Invitrogen). The membrane was blocked in 5% skimmed milk powder (Marvel) in TBS 0.1% volume for volume Tween20 for 1 hour at room temperature. The primary antibodies (p53 1:100 IS616 Dako, β-actin 1:2000 A1978, RRID:AB_476692, Sigma, TGFBI 1:500 60007-1-Ig, RRID:AB_10896828, Proteintech) were diluted in blocking buffer and incubated overnight at 4°C. The membrane was incubated with horseradish peroxidase (HRP)-conjugated antibody (Anti-mouse 1:2000 NXA931, RRID:AB_772209, GE Healthcare) for 1 hour at room temperature. HRP activity was visualised with Amersham ECL (RPN2232, GE Healthcare) or Luminata Forte (WBLUF0500, Millipore) and Amersham Imager 600 (GE Healthcare).

### ELISA

The human TGFBI ELISA (Thermo Fisher Scientific, EHTGFBI) was performed according to manufacturer's instructions. Supernatants were diluted 1:500 to ensure TGFBI detection within the range of the standard curve. Abs_450_ was read using a BMG Labtech FLUOstar Optima reader (Labtech). TGFβ1 And TGFβ2 ELISA were from RnD Biosystems (DY240-05, DB250) and were performed according to manufacturer's instructions.

### Mouse experiments

Mouse experiments were performed under the license PBE3719B3 in accordance with Animals (Scientific Procedures) Act 1986 with the approval of our Institutional Ethics committee. Six-week-old C57BL/6NCrl female mice, RRID:IMSR_CRL:027, were purchased from Charles River. Mice received 1 × 10^7^ HGS2 cells injected intraperitoneally in 300 μL PBS as described previously (17). Mice were treated intraperitoneally with anti-TGFBI antibody (26, 27) or mouse IgG1 isotype MOPC-21, RRID:AB_1107784 (Bioxcell), 300 mg/kg, starting week 7, twice weekly for 3 weeks.

## Results

### Upregulation of TGFBI in STIC lesions and primary HGSOC stroma

**Figure 1.**
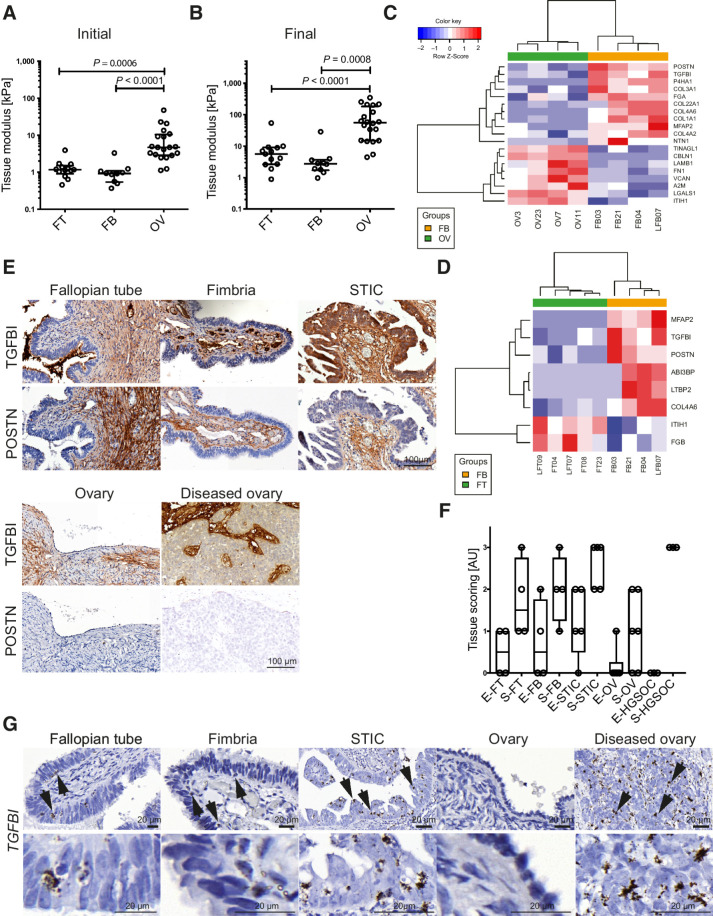
Identification of tissue modulus and matrisome proteins that define tissue architecture of human OV and FT tissues. **A** and **B,** Initial and final tissue modulus of human FT, FB, and OV tissues. Statistical significance was determined using Kruskal–Wallis test with Dunn multiple comparison test. **C** and **D,** Heatmap depicting matrisome proteins differentially expressed between OV (*n* = 4), FB (*n* = 4), FT (*n* = 5), and FB tissues. **E,** IHC of TGFBI and POSTN. Representative images from FT (*n* = 4), FB (*n* = 4), STIC (*n* = 7), ovary (*n* = 6), and diseased ovary (*n* = 3) tissues. **F,** Modified Allred scoring of the matrisome protein TGFBI. Scoring describes the percentage of positive staining (0 = negative, 1 = weak, 2 = moderate, 3 = strong). Scoring was performed on epithelial (E) and stromal (S) areas of FT (*n* = 4), FB (*n* = 4), STIC (*n* = 5), ovary (*n* = 6), and diseased ovary (*n* = 3) tissues. **G,** ISH of *TGFBI* in healthy and diseased FT and OV tissues. Arrows indicate cells with a high copy number of *TGFBI* mRNA and cytoplasmic projections. Representative images of FT (*n* = 3), FB (*n* = 3), STIC (*n* = 9), ovary (*n* = 4), and invasive HGSOC at the ovary (*n* = 6).

We analyzed the mechanical properties and ECM of fresh, healthy tissues from human FT, FB (the most distal part of the FT and the location of STIC lesions), and OV tissue. Our aim was to compare the stiffness of the tissues and to identify differentially expressed matrix proteins between FT and FB or OV, which may be involved in driving transformation and metastasis. We used fresh human tissue to compare the proximal FT and OV with the healthy, distal FB (because the majority of STIC lesions require microscopic examination to be identified in an otherwise grossly normal-appearing FB). Mechanical indentation was used to determine tissue modulus, which describes material stiffness independent of sample dimension and the stress relaxation behavior of the samples. The initial (2.5%–7.5%) tissue modulus of the FT and FB was approximately 1 kPa, whereas OV reached median values around 4 kPa (Fig. 1A). The final (25%–30%) tissue modulus was approximately 6 and 3 kPa for the FT and FB, while the healthy and diseased human OV had a final tissue modulus of 56 and 48 kPa, respectively (Fig. 1B). Therefore, transformed FT surface epithelial, FTSE, cells experience a large increase in tissue stiffness when metastasizing to the ovary.

As there was a magnitude of difference in tissue modulus between the FT and FB compared with OV tissues, we next assessed whether there was a change in the expression of ECM proteins. Whole tissue lysates were enriched for the matrisome protein fraction and analyzed via mass spectrometry. Differential expression analysis revealed increased POSTN and TGFBI abundance in the FB in comparison with the OV, but also in comparison with the proximal FT, indicating that these matrisome proteins may be found in the microenvironment where STIC lesions are found (Fig. 1C and D).

To confirm the matrisome data, we assessed POSTN and TGFBI by immunohistochemistry of STICs and diseased OV (Fig. 1E and F; Supplementary Fig. S1). POSTN protein was confined to the stroma, whereas TGFBI was found in STIC epithelium and stroma at increased levels compared to healthy FB and in the stroma of diseased OV tissue. As there were published data showing that TGFBI can be produced by fibroblasts and peritoneal cells, and may aid the spread of ovarian cancer cells throughout the peritoneal cavity by increasing their adhesive, mobile, and invasive potential (28, 29), we focused on TGFBI, conducting RNA scope ISH on healthy and diseased FT, FB, and OV. There were few *TGFBI* transcripts in the healthy epithelium, STIC cells and malignant cells. However, there was a distinct population of cells with a strong signal adjacent to diseased epithelium of STIC lesions and to the malignant cells in the ovary (Fig. 1G).

### CD163^+^ macrophages produce TGFBI in STIC lesions and HGSOC

**Figure 2.**
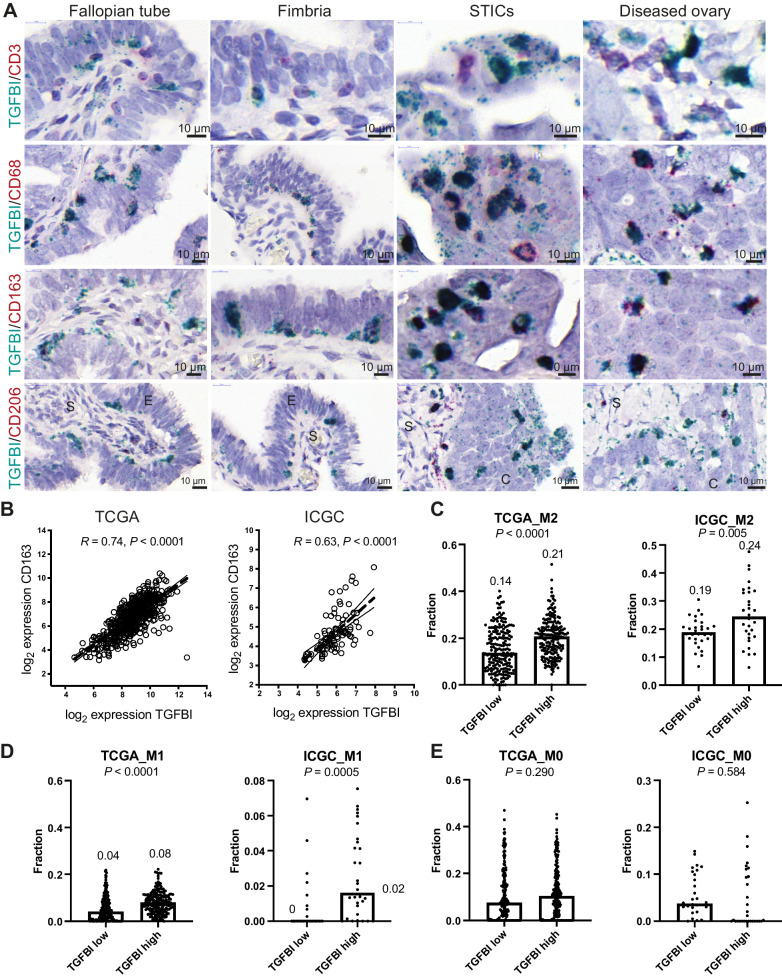
TGFBI is produced by TAMs. **A,** Dual *ISH* for TGFBI with either CD3, CD68, CD163, or CD206 in healthy and diseased FT and OV. **B,** Correlation of CD163 with TGFBI within TCGA and ICGC HGSOC transcriptional datasets. **C–E,** CIBERSORT fractions of M2, M1, and M0 macrophages in TGFBI low and high patients of TCGA and ICGC dataset. Statistical significance was determined using Student *t* test. Median values are indicated.

The cells with the strongest *TGFBI* signal had the morphologic appearance of immune cells. Therefore, we carried out dual RNA scope with immune cell markers *CD3* (lymphocytes) or *CD68* (macrophages) and *TGFBI* (Fig. 2A). There was no colocalization of *CD3* mRNA with *TGFBI* but *CD68* and *TGFBI* mRNA colocalized in all tissues. *CD163* and *CD206* are often expressed on alternatively activated macrophages and these also colocalized with *TGFBI* transcripts. *CD206* positivity was only observed in stromal *TGFBI*-expressing macrophages whereas *CD163* colocalized with macrophages in all locations where *TGFBI* expression was found (Fig. 2A).

We then interrogated publicly available HGSOC transcriptional datasets to see whether there was an association between TGFBI expression and TAMs in primary tumors. There was a strong correlation between *TGFBI* and *CD163* expression in TCGA (*R* = 0.74, *P* < 0.0001) and ICGC (*R* = 0.63, *P* < 0.0001) datasets (Fig. 2B). Analysis of the 30% highest and 30% lowest *TGFBI*-expressing tumors in the TCGA and ICGC datasets using CIBERSORT (22) revealed a significantly increased proportion of alternatively activated (“M2”) macrophages in the *TGFBI* high-expressing tumors (Fig. 2C; Supplementary Table S2). The “M1” fraction of classically activated macrophages represented a considerably smaller fraction of the immune infiltrate, and was also significantly increased in the *TGFBI* high-expressing tumors (Fig. 2D; Supplementary Table S2). This association was not found with the M0 fraction (Fig. 2E; Supplementary Table S2).

In summary, both ISH of human tissues and gene expression data suggest a strong association of *TGFBI* expression and macrophages in ovarian cancer; this correlation is present in the very early stages of the disease in the FB but also in HGSOC cancers in the ovary.

### TGFBI expression and secretion by macrophages *in vitro*

**Figure 3.**
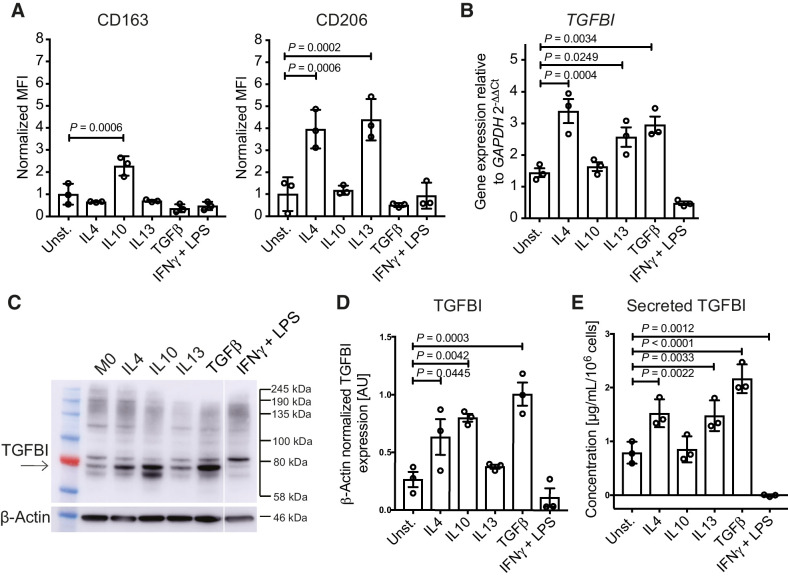
TGFBI expression and secretion by macrophages *in vitro*. **A,** CD163 and CD206 surface expression, determined by flow cytometry, in human monocyte-derived macrophages stimulated with the cytokines IL4, IL10, IL13, TGFβ, and IFNγ+LPS. Data (*n* = 3) shown are mean ± SD. Statistical significance was determined using one-way ANOVA with Dunnett multiple comparisons test. MFI, mean fluorescence intensity. **B,***TGFBI* expression of monocyte-derived macrophages stimulated for 3 days. Data (*n* = 3) shown are mean ± SD. Statistical significance was determined using one-way ANOVA with Dunnett multiple comparisons test. **C** and **D,** Western blot analysis and quantification of TGFBI in cytokine-stimulated monocyte-derived macrophages. TGFBI band is indicated by the arrow. **E,** Secreted TGFBI levels by macrophages stimulated for 3 days measured by ELISA. Data (*n* = 3) shown are mean ± SD. Statistical significance was determined using one-way ANOVA with uncorrected Fisher LSD multiple comparisons test.

To further confirm macrophages as a source of TGFBI, we stimulated human monocyte-derived macrophages with Th1 (IFNγ+LPS) and Th2 (IL4, IL10, IL13, and TGFβ) cytokines *in vitro*. After 7 days differentiation, macrophages were stimulated with these mediators for 3 days and stained for CD163 and CD206 (Fig. 3A, gating in Supplementary Fig. S2A). All populations were positive for both markers, IL10 significantly upregulated CD163 and both IL4 and IL13 upregulated CD206. Using quantitative PCR, we observed a significant increase *TGFBI* expression in IL4, IL13, and TGFβ-stimulated macrophages (Fig. 3B). Western blotting showed that IL4, IL10, and TGFβ significantly induced TGFBI protein in macrophage lysates (Fig. 3C and D).

To investigate whether the stimulated macrophages secreted TGFBI protein, we performed ELISA using conditioned medium from stimulated macrophages. TGFBI secretion was significantly increased when macrophages were stimulated with IL4, IL13, and TGFβ (Fig. 3E). In contrast, IFNγ+LPS treatment significantly reduced TGFBI secretion. Supplementary Table S3 summarizes these data. We conclude that *in vitro* there was increased production of TGFBI by macrophages stimulated by cytokines with immunosuppressive actions and decreased production of TGFBI by cytokines associated with antitumor responses.

### Mutant p53 FTSE cells stimulate macrophages to produce TGFBI

**Figure 4.**
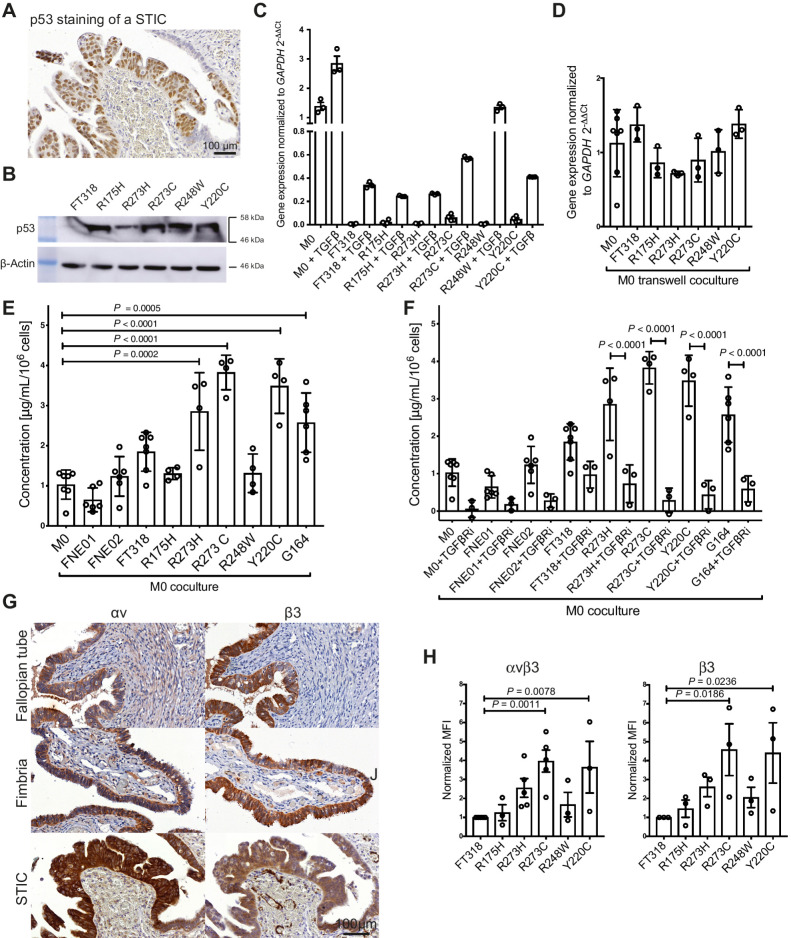
Stimulation of TGFBI expression in macrophages by p53 mutated FTSE cells resembling STICs. **A,** Expression of p53 in STIC lesions. **B,** Expression of p53 in FT318 wild-type and mutant p53 transduced FTSE cells (R175H, R273H, R273C, R248W, Y220C). β-actin was used as loading control. **C,***TGFBI* transcript expression of unstimulated and TGFβ-stimulated FT318 wild-type and mutant p53 FTSE cell lines in comparison with unstimulated (unst.) and TGFβ-stimulated macrophages. Data (*n* = 3) shown are mean ± SEM. Statistical significance was determined using one-way ANOVA with Dunnett multiple comparisons test. **D,***TGFBI* expression in macrophages cultured in transwells with FTSE wild-type and FTSE-mutant cell lines. Data are shown for unstimulated macrophages (*n* = 7), FT318 (*n* = 3), R175H (*n* = 3), R273H (*n* = 3), R273C (*n* = 3), R248W (*n* = 3), and Y220C (*n* = 3). Statistical significance was determined using one-way ANOVA with Dunnett multiple comparisons test.**E,** TGFBI secretion of macrophages alone (M0) or cocultured with FTSE cell lines [wild type (FNE01, FNE02, FT318) and mutant p53 (R175H, R273H, R273C, R248W, Y220C)] and a HGSOC cell line (G164) normalized for 10^6^ macrophages. Data are mean ± SD. Macrophages (*n* = 7), FNE01 (*n* = 6), FNE02 (*n* = 6), FT318 (*n* = 7), R175H (*n* = 4), R273H (*n* = 4), R273C (*n* = 4), R248W (*n* = 4), Y220C (*n* = 4), G164 (*n* = 6). Statistical significance determined using one-way ANOVA with Dunnett multiple comparisons test. Compared with coculture with FT318, secretion of TGFBI is significant only for cell lines R273C and Y220C. **F,** TGFBI secretion of cocultured macrophages with FTSE and HGSOC cells in the presence or absence of the selective TGFβR inhibitor SB431542. Data are mean ± SD. *n* = 3 for all the inhibitor-treated conditions. Statistical significance was determined using one-way ANOVA with Dunnett multiple comparisons test. Untreated coculture data points are the same as in **E**. **G,** IHC staining of healthy and diseased human tissues for the integrin subunits αv and β3. Representative data are shown for FT (*n* = 3), FB (*n* = 3), STICs (*n* = 7), ovary (*n* = 3), and HGSOC diseased ovary (*n* = 4) tissues. **H,** FT318 wild-type and mutant p53 (R175H, R273H, R273C, R248W, Y220C) FTSE cells stained for the integrin αvβ3 [*n* = 5 (FT318, R273C, R273H); *n* = 3 (R175H, R248W, Y220C)] and the subunit β3 (*n* = 3). Data are mean ± SEM. Statistical significance determined using one-way ANOVA with Bonferroni multiple comparisons test. MFI, mean fluorescence intensity.

As *TGFBI* was expressed at low levels by STIC epithelial cells in patient tissues and TGFBI protein was present on these cells (Fig. 1E), we next compared *TGFBI* expression by macrophages and FTSE cells *in vitro*. Gain of function (GOF) *TP53* mutations are a crucial part of the transition of a normal cell into a STIC cell in HGSOC (Fig. 4A; ref. 30) and almost all HGSOCs harbor a *TP53* mutation (31–33). We used immortalized human FTSE cells (FT318) with a range of *TP53* hotspot mutations (R175H, R273H, R273C, R248W, Y220C) that are associated with a GOF phenotype in ovarian tumors (Fig. 4B). When comparing mRNA levels between FTSE cells and unstimulated macrophages, a significant (*P* < 0.0001) fold change difference was observed, again suggesting that macrophages are the predominant producers of *TGFBI* (Fig. 4C). Moreover, FTSE cells did not secrete any TGFBI protein (Supplementary Fig. S2B). However, stimulation of FTSE cells with TGFβ increased *TGFBI* mRNA expression, albeit to a significantly lower extent (*P* < 0.0001) than in macrophages, with the exception of the FT318-mutant p53 R248W cells, which reached, upon TGFβ stimulation, TGFBI mRNA levels similar to unstimulated macrophages. All other FTSE cells had significantly lower *TGFBI* mRNA levels in comparison with unstimulated macrophages (Fig. 4C).

To investigate whether FTSE cells with wild-type or mutant p53 can stimulate macrophages to produce TGFBI, macrophages were co-cultured in a transwell system with FT318 wild-type or mutant p53 cells. Wild-type and mutant p53 FTSE cells did not induce macrophages to produce TGFBI when they were not in direct contact (Fig. 4D). However, when mutant p53 R273H, R273C, and Y220C FTSE cells were directly cocultured with macrophages, there was a significant increase in secreted TGFBI protein (Fig. 4E). Wild-type p53 cell lines FT318, FNE01, and FNE02, and the other p53-mutant cell lines, did not induce significant increase of TGFBI secretion. A malignant HGSOC cell line, G164 (25), upregulated TGFBI secretion by macrophages in a similar fashion to the mutant p53 FTSE cells (Fig. 4E).

To see whether FTSE mutant p53 and HGSOC cell stimulation of macrophage TGFBI production involves TGFβ signaling through the TGFBR, a selective TGFBR type 1 activin receptor-like kinase (ALK5) inhibitor, SB431542, was used (Fig. 4F). SB431542 significantly inhibited *TGFBI* mRNA transcript production in the FT318 mutant p53 as well as G164 HGSOC cell-macrophage cocultures, suggesting TGFβ signaling is required for the stimulation of TGFBI expression in macrophages. However, there was no change in macrophage phenotype in any of the cocultures as assessed by flow cytometry for CD163 and CD206 (Supplementary Fig. S2C). Supernatants from this experiment were tested for TGFβ1 and TGFβ2 protein secretion by ELISA, but TGFβ levels did not correlate with TGFBI levels (Supplementary Fig. S2D), suggesting that more complex mechanisms might be at play. Interestingly, we found that integrin αvβ3, a putative cell surface receptor for TGFBI (34), which can also regulate TGFβ activity, is expressed on FTSE cells of healthy FT and FB tissue, as well as STIC epithelium (Fig. 4G). Furthermore, αvβ3 levels were higher in two of the mutant p53 FTSE cell lines that induced macrophage-derived TGFBI secretion at the highest levels, R273C and Y220C, compared with normal FT lines (Fig. 4H).

Taken together, these data show that some mutant FTSE cells and malignant HGSOC cells were able to induce TGFBI production by macrophages *in vitro*.

### TGFBI secretion and macrophage stimulation by cancer-associated fibroblasts in HGSOC biopsies

**Figure 5.**
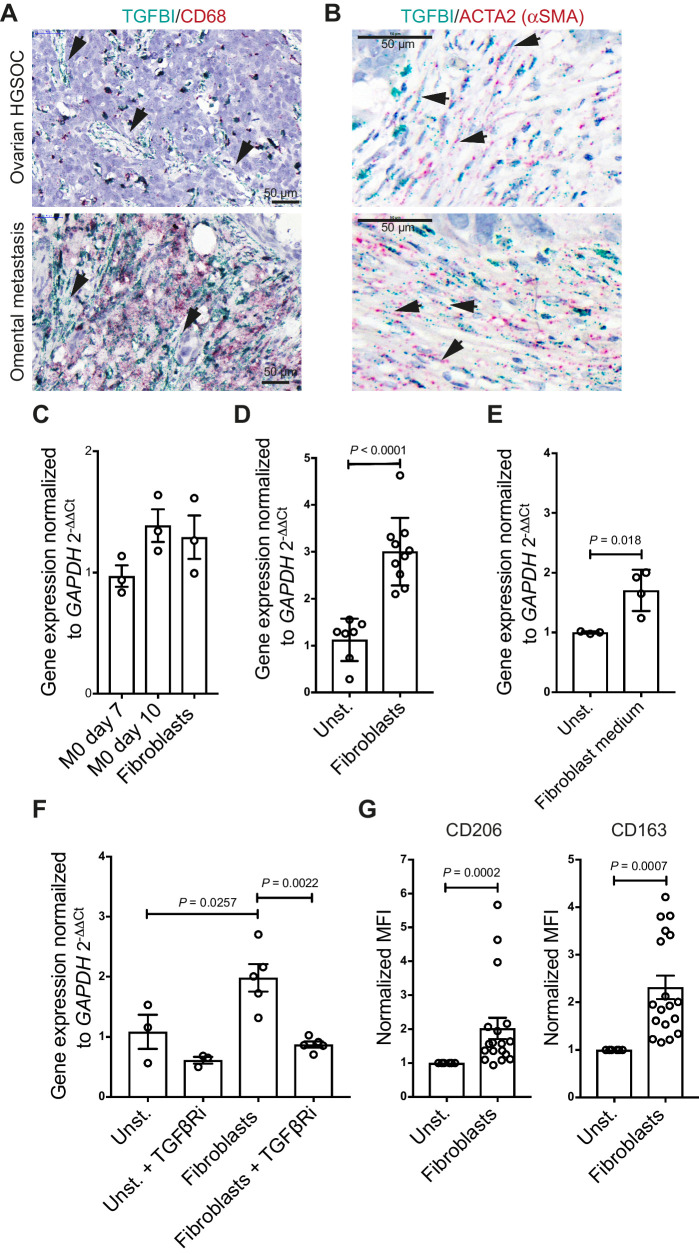
Cross-talk between TGFβ-secreting fibroblasts and macrophages. **A,** Dual ISH for TGFBI and CD68 in HGSOC in the ovary and omentum. Fibroblast-like cells (black arrows) span the stroma and express TGFBI but not CD68. **B,** Dual ISH for TGFBI and ACTA2 in HGSOC in the ovary and omentum. Examples of ACTA2-TGFBI are indicated by black arrows. **C,***TGFBI* mRNA expression comparing the basal levels of transcript in unstimulated macrophages (*n* = 3) and primary omental fibroblasts (*n* = 3). Statistical significance was determined using one-way ANOVA with Dunnett multiple comparisons test. Nonsignificant, *P* > 0.05. **D,***TGFBI* mRNA expression of cocultured macrophages with primary fibroblasts. Data shown are mean ± SD. Unstimulated macrophages, *n* = 7; fibroblasts, *n* = 10. Statistical significance was determined using one-way ANOVA with Dunnett multiple comparisons test. **E,***TGFBI* mRNA expression by macrophages cultured in 50% fresh culture medium and 50% fibroblast-derived medium. Data shown are mean ± SD. Unstimulated (*n* = 3) versus fibroblast medium of four different donors (*n* = 4). Statistical significance was determined using unpaired *t* test. **F,***TGFBI* mRNA expression of cocultured macrophages with omental fibroblasts in the presence or absence of the selective TGFβRi. Data shown are mean ± SD. *n* = 3 and *n* = 4 for unstimulated macrophages and macrophage/fibroblast cocultures (matched untreated and TGFβRi treated), respectively. Statistical significance was determined using one-way ANOVA with Dunnett multiple comparisons test. **G,** CD206 and CD163 expression post coculture with primary fibroblasts. Data were normalized to unstimulated macrophages. Data shown are mean ± SD. Unstimulated macrophages (*n* = 3) versus macrophages (two different peripheral blood mononuclear cell donors) cocultured with fibroblasts of up to seven different fibroblast donors (*n* = 11). Statistical significance was determined using unpaired *t* test. MFI, mean fluorescence intensity.

Although the majority of *TGFBI* transcripts in more advanced HGSOC biopsies and late-stage omental metastasis were localized in macrophages, some cells with fibroblast-like morphology also expressed *TGFBI* (Fig. 5A). We further investigated this and, in accordance with our findings in pancreatic cancer biopsies and mouse pancreatic cancer models (27), we also identified *TGFBI* transcript in some of the *ACTA2*-expressing fibroblasts in the stroma of ovarian and omental metastasis (Fig. 5B). We studied *TGFBI* expression in primary omental fibroblasts from HGSOC donor tissues that spanned a wide range of disease burden, from uninvolved omentum through to metastatic HGSOC. Similar levels of *TGFBI* transcripts were expressed by differentiated monocyte-derived macrophages and omental fibroblasts (Fig. 5C) and omental fibroblasts significantly (*P* < 0.0001) stimulated macrophages in transwell cocultures to produce *TGFBI*, irrespective of whether the fibroblasts had originated from diseased or uninvolved omentum (Fig. 5D). Furthermore, fibroblast-conditioned medium induced *TGFBI* expression in macrophages, indicating that unlike for the FTSE and HGSOC cells, soluble factors from fibroblasts were sufficient to induce expression (Fig. 5E). However, when compared with the transwell coculture, the increase in expression was less pronounced (1.5-fold vs. 3-fold).

SB431542A significantly inhibited *TGFBI* mRNA transcript production in the fibroblast-macrophage transwell cocultures, suggesting TGFβ signaling is also crucial for the fibroblast stimulation of TGFBI expression in macrophages (Fig. 5F). Furthermore, CD163 and CD206 were significantly upregulated when macrophages were co-cultured with fibroblasts (Fig. 5G). The ability of fibroblasts to induce the upregulation of markers indicative of alternative activation of macrophages may be due to their culture on tissue culture plastic prior to the transwell coculture. Tissue culture plastic has a stiffness in the range of giga Pascals, and thus may activate fibroblasts, suggesting that all fibroblasts have been activated to some degree, irrespective of the donor from which they originated (35).

We therefore suggest a mechanism by which transformed FTSE cells, and at later disease stages activated fibroblasts, induce the expression of *TGFBI* in macrophages, in part through the secretion of TGFβ. Once the secretory cells of the epithelium acquire a *TP53* mutation, they may upregulate integrins, such as αvβ3, that allows them to bind to overexpressed matrix proteins, such as TGFBI. Also, IL4 secreted by Th2 T cells may play a role in the secretion of TGFBI by macrophages, which may be mediated in an autocrine TGFβ-dependent fashion (36). Therefore, TGFβ signaling in the FB may prime macrophages to secrete TGFBI, which is an effector of an immunosuppressive microenvironment promoting transformed FTSE cell growth and STIC development.

If TGFBI is involved in immune suppression and is also found in advanced tumors, we hypothesized that it may contribute to the immune landscape of advanced HGSOC tumors, in line with our previously reported results in a mouse pancreatic cancer model (27).

### Inhibition of TGFBI in a HGSOC *in vivo* model reduces tumor growth

**Figure 6.**
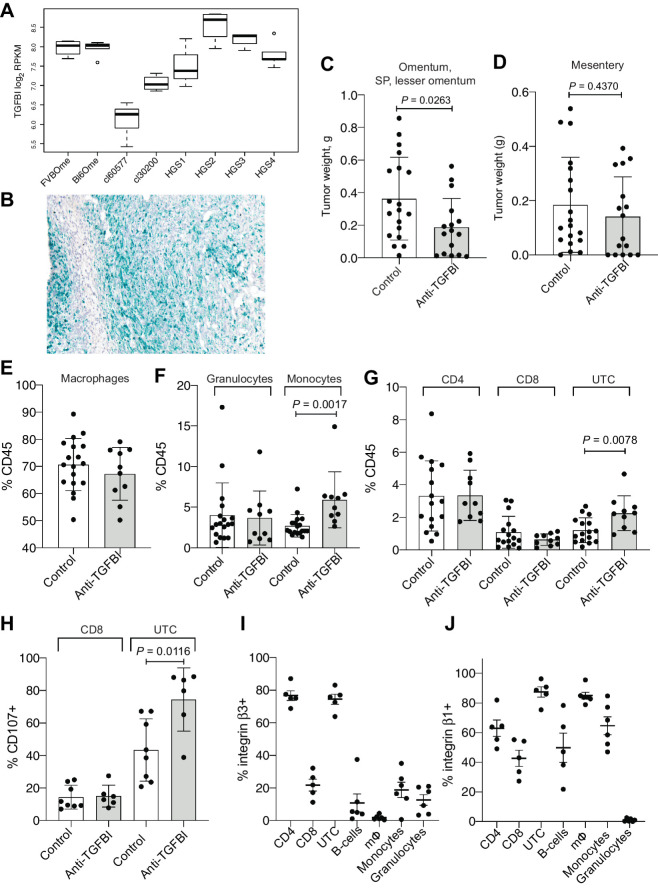
Effect of anti-TGFBI treatment in an *in vivo* model of HGSOC. **A,** Boxplot of *Tgfbi* gene expression in log_2_ read counts per million (RPKM) across healthy omentum of FVB (*n* = 4) and C57BL6 mice (*n* = 5) and omental tumors from the 30200 (*n* = 4), 60577 (*n* = 5), HGS1 (*n* = 3), HGS2 (*n* = 4), HGS3 (*n* = 4), HGS4 models (*n* = 5) from GSE132289. **B,** RNA-scope for TGFBI in an omental tumor from the HGS2 model. **C** and **D,** Omentum, SP, LO (**C**) and mesenteric (**D**) tumor weight for mice injected with HGS2 and treated for 3 weeks with anti-TGFBI, starting at week 7 (*n* = 18 for controls; *n* = 10 for anti-TGFBI treated). Statistical significance was determined using unpaired *t* test. **E–G,** Percentage of macrophages, granulocytes, monocytes, CD4, CD8, and UTC among CD45-positive cells in omental tumors from control-treated and anti-TGFBI–treated mice (*n* = 18 for controls; *n* = 10 for anti-TGFBI treated). Statistical significance was determined using *t* test. **H,** Percentage of CD8 and UTC cells positive for CD107a (surface staining) in omental tumors from control-treated and anti-TGFBI–treated mice. (*n* = 8 for controls; *n* = 6 for anti-TGFBI treated). Statistical significance was determined using *t* test. **I** and **J,** Percentage of cells positive for integrin β3 and β1subunits staining on different populations of immune cells infiltrating untreated omental tumors generated by injecting HGS2 (percentage of integrin-positive cells for each population, from *n* = 5 tumors).

To investigate this hypothesis, we targeted TGFBI in one of our transplantable orthotopic mouse models of HGSOC, HGS2, that replicates many of the molecular and cellular features of the human disease (17). HGS2 is a *Trp53^−/−^*, *Pten*, and *Brca-2^−/−^* cell line and was generated from tumors derived from a genetic model established by Perets and colleagues (4) and backcrossed to B6 mice. Bulk RNA-sequencing analysis showed that established HGS2 models had high levels of TGFBI (Fig. 6A), and this was confirmed by RNAscope (Fig. 6B).

Mice were treated with anti-TGFBI antibody (26, 27) twice a week for 3 weeks starting at week 7 after intraperitoneal injection of HGS2 cells (a time when tumors were established). We observed a significant reduction of the cumulative weight at the omental, splenoportal and lesser omentum sites compared with the control group (Fig. 6C; Supplementary Fig. S3A). However, no effect was seen on the tumors developing in the mesentery (Fig. 6D). We previously reported that inhibiting TGFBI resulted in a remodeling of the pancreatic tumor microenvironment with an enhanced antitumor response (27). In the HGS2 tumors, however, we observed an increase of the percentage of monocytes infiltrating the tumors, but the proportion of macrophages and granulocytes was unchanged (Fig. 6E and F; gating strategy is shown in Supplementary Fig. S3B and S3C). There was also no change in expression of “M1” markers CD86 and MHCII although there was a significant increase in CD206^+^ cells (Supplementary Fig. S3D). The percentage of CD4^+^ and CD8^+^ lymphocytes was not affected by anti-TGFBI treatment, but the percentage of unconventional T cells (UTC; gated as CD45^+^ CD3^+^ CD4*^−^* CD8*^−^*) increased significantly (Fig. 6G). Differently from CD4^+^ and CD8^+^ lymphocytes, UTCs target monomorphic Ag-presenting molecules and other ligands. This includes the MHC class 1b–restricted T cells, CD1- and MR1-restricted T cells, and gamma delta T cells (37). Interestingly, the UTCs also exhibited increased activation in tumors from treated mice, as shown by a higher proportion of UTCs positive for CD107a surface staining. CD8 activation levels were unchanged (Fig. 6H). This is in contrast to the pancreatic cancer model, where anti-TGFBI treatment increased the number and activation of CD8^+^ cells.

We next analyzed the expression of integrin β3, one of the main integrin subunits responsible for TGFBI binding (34), on the immune infiltrate of nontreated omental tumors. The majority of UTCs expressed integrin β3, while only a small proportion of CD8^+^ cells were positive for this integrin (Fig. 6I). CD4^+^ cells were also highly positive for integrin β3 expression (Fig. 6I). We also investigated integrin β1 expression, as it is also known to mediate binding to TGFBI (38). Again, UTCs were highly positive for integrin β1 expression, together with macrophages (Fig. 6J). A proportion of CD8^+^ cells were positive for integrin β1, although it was still lower than the integrin β1–positive UTCs (Fig. 6J).

**Figure 7.**
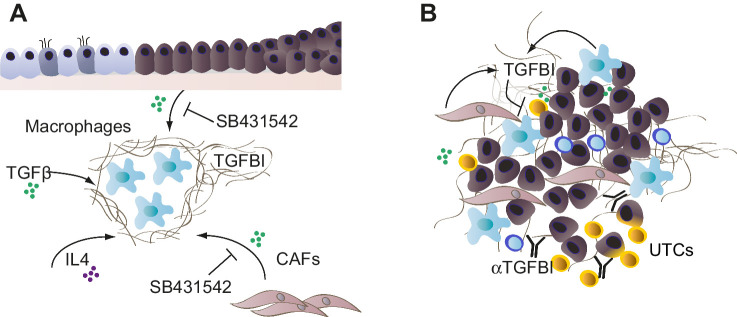
Potential actions of TGFBI in STICs and advanced HGSOC. **A,** In the early stages of transformation, FTSE cells in the FB induce TGFBI in macrophages, in part through the secretion of TGFβ. Once the secretory cells of the epithelium acquire a *TP53* mutation, they may further upregulate integrins, such as αvβ3, that allows them to bind to overexpressed matrix proteins, such as TGFBI. IL4 secreted by Th2 T cells may play a role in the secretion of TGFBI by macrophages. Therefore, TGFβ signaling in the FB may prime macrophages to secrete TGFBI, which is an effector of an immunosuppressive microenvironment promoting transformed FTSE cell growth and STIC development. **B,** In established HGSOC tumors, TGFBI is produced by macrophages, and fibroblasts especially interact with unconventional T cells, UTCs in omental metastases. Anti-TGFBI antibodies stimulate UTC infiltration and activation in the tumor microenvironment.

In conclusion, inhibition of TGFBI in an HGSOC model reduced peritoneal tumor size. This was linked to changes in the tumor immune microenvironment, in particular with an increase of monocytes and UTCs. UTCs were also activated at higher levels by anti-TGFBI. As these cells express high integrin levels responsible for the binding of TGFBI, we hypothesize that TGFBI has an inhibitory action on these cells, and activation can be enhanced by blocking TGFBI. In Fig. 7 we have summarized all our findings on the actions of TGFBI in STICs (Fig. 7A) and advanced HGSOC (Fig. 7B).

## Discussion

In this study, we have provided evidence that the ECM protein TGFBI (βig-h3) contributes to an immune-suppressive microenvironment in HGSOC. Our study began with a finding in the earliest lesions of this disease and extended to advanced disease in patient biopsies and a mouse HGSOC model.

There is limited information on the role of TGFBI in malignant disease, especially in the context of immune responses. TGFBI is a 68 kDa matricellular protein implicated in cell–matrix interactions and cell migration (39). There is evidence of TGFBI binding to ECM proteins such as fibronectin, SPARC (40), and several collagens but it is not thought to be a structural protein *per se*. In macrophage:fibroblast coculture experiments, ingestion of apoptotic cells stimulated collagen protein production by fibroblasts and this was mediated by TGFBI (41). This interaction could contribute to resolution of inflammation and wounds but if dysregulated during pathologic processes, could contribute to the abnormal fibrosis seen in malignancy and chronic tissue damage, which is of particular interest given the importance of fibrosis in inhibiting immune response. Other information on the role of TGFBI in cancer generally points to a tumor-promoting role, although *Tgfbi* null mice, which have retarded growth, also have an increased incidence of spontaneous and carcinogen-induced cancers (42). Elevated levels of TGFBI have been associated, in common with its paralogue POSTN, with poor survival in patients with ovarian cancer (43).

TGFBI may be involved in cell migration in cancer. Loss of TGFBI alters microtubule stability and may impair cell mobility (44). TGFBI preferentially interacts with cells through an ανβ3 integrin-mediated mechanism (45) and loss of TGFBI is sufficient to induce specific resistance to paclitaxel by altering microtubule stabilization via integrin-mediated activation of focal adhesion kinase and Rho family GTPase (46). Apart from confirming the association between of high levels of TGFBI and poor prognosis, Steitz and colleagues found that ovarian cancer cell migration was stimulated by soluble mediators produced by macrophages from HGSOC ascites as well as IL10-stimulated monocyte-derived macrophages. They identified three proteins from these macrophage secretomes that could stimulate cancer cell migration, one of which was TGFBI. Neutralizing antibodies and siRNA partially abolished the migration inducing activity of TGFBI. The Steitz and colleagues article supports our finding of macrophage production of TGFBI and also our suggestion that it may be involved in early stages of transformed FTSE cell migration to the ovary.

In addition, our work suggests a role for TGFBI in the HGSOC immune microenvironment. The first report to demonstrate that TGFBI acts on tumor immune cells came from studies in pancreatic cancer (27). Investigating high levels of fibroblast TGFBI in pancreatic cancer tissues, Goehrig and colleagues found that this molecule interacted directly with T cells and macrophages and that neutralising anti-TGFBI antibodies reduced tumor growth, enhanced CD8^+^ T-cell activation, and polarized macrophages to an M1 state. In the current article, short-term treatment of advanced peritoneal metastases also reduced tumor growth compared with controls with changes to the immune microenvironment, but these were different to those reported in the pancreatic cancer models. In our HGSOC model, CD8^+^ lymphocytes were not affected but we observed an increase in, and activation of, UTCs. This could be explained by a different pattern of integrin expression in our model. While in pancreatic tumors, CD8 cells expressed high levels of integrin b3 (27), we found that UTCs express higher levels of integrin β3 and β1 in the omental HGSOC tumors compared with CD8^+^ cells. Interestingly, some classes of UTCs are highly represented in human omentum (47) but reduced in cancer and obesity. This higher abundance of UTCs in the omentum might also explain our findings. UTCs are characterized by the expression of different classes of invariant forms of the T-cell receptor, TCR, and can play a prominent antitumor role (37). Our results imply that one or more UTC subtypes are activated and degranulate with anti-TGBI treatment, suggesting they had direct cytotoxic activity on malignant cells.

There is little information on the role of TGFBI in immunity. TGFBI mRNA levels are elevated in human lymphoid tissues, high levels were detected in immature dendritic cells and were able to stimulate macrophage endocytosis, leading Cao and colleagues to speculate a role for TGFBI in immune regulation (48). Under conditions that mimic low antigen stimulation, TGFBI can also inhibit CD8^+^ cell responses through inhibition of TCR signaling (49).

Although it is thought that the abnormal ECM in cancers can impact on immune cell infiltrates and access to drugs, therapies that target ECM production have not yet fulfilled their potential. The HGSOC study reported here, taken together with the findings in pancreatic cancer, would suggest that approaches that target TGFBI are worthy of further investigation. Possibly this ECM protein is another downstream mediator of the well-documented immunosuppressive actions of TGFβ?

## Authors' Disclosures

L.S.M. Lecker reports employment with Novartis since January 2019. J.D. Brenton reports being a cofounder and shareholder of Tailor Bio. P.R. Cutillas reports grants from Cancer Research UK during the conduct of the study and personal fees from Kinomica Ltd outside the submitted work. R. Drapkin reports personal fees from Repare Therapeutics, Cedilla Therapeutics, Boehringer Laboratories, and other support from VOC Health outside the submitted work. F.R. Balkwill reports personal fees from Verseau Therapeutics Inc., Novartis Inc., GlaxoSmithKline, and Mestag Therapeutic Ltd. during the conduct of the study. No disclosures were reported by the other authors.

## Supplementary Material

Table S1 S2 S3 legend Legend of supplementary tables

Figure S1 IHC of TGFBI and POSTN

Figure S2 Effect of macrophages and FTSE and HGSOC cell lines co-culture on CD163 and CD206 expression

Figure S3 Effect of anti-TGFBI treatment in the HGS2 model

Table S1 List of antibodies used for flow cytometry

Table S2 CIBERSORT results of the TGFBI high/low samples in the TCGA and ICGC datasets

Table S3 Summary of TGFBI expression and secretion by stimulated macrophages
