# Supplementary material for: TGFBI Production by Macrophages Contributes to an Immunosuppressive Microenvironment in Ovarian Cancer
Source: Cancer Res. 2021 Sep 24;81(22):5706–19. doi: 10.1158/0008-5472.CAN-21-0536 (PMC9397609; doi:10.1158/0008-5472.CAN-21-0536)
Supplement: Table S1 S2 S3 legend — Legend of supplementary tables [file can-21-0536_table_s1_s2_s3_legend_suppstl1-stl3.docx]

Supplementary Table 1: List of antibodies used for flow cytometry

Supplementary Table 2: CIBERSORT results of the TGFBI high/low samples in the TCGA and ICGC datasets.

Supplementary Table 3: Summary of TGFBI expression and secretion by stimulated macrophages.
