# Supplementary material for: TGFBI Production by Macrophages Contributes to an Immunosuppressive Microenvironment in Ovarian Cancer
Source: Cancer Res. 2021 Sep 24;81(22):5706–19. doi: 10.1158/0008-5472.CAN-21-0536 (PMC9397609; doi:10.1158/0008-5472.CAN-21-0536)
Supplement: Figure S1 — IHC of TGFBI and POSTN [file can-21-0536_figure_s1_suppsf1.pdf]

Supplementary Figure 1

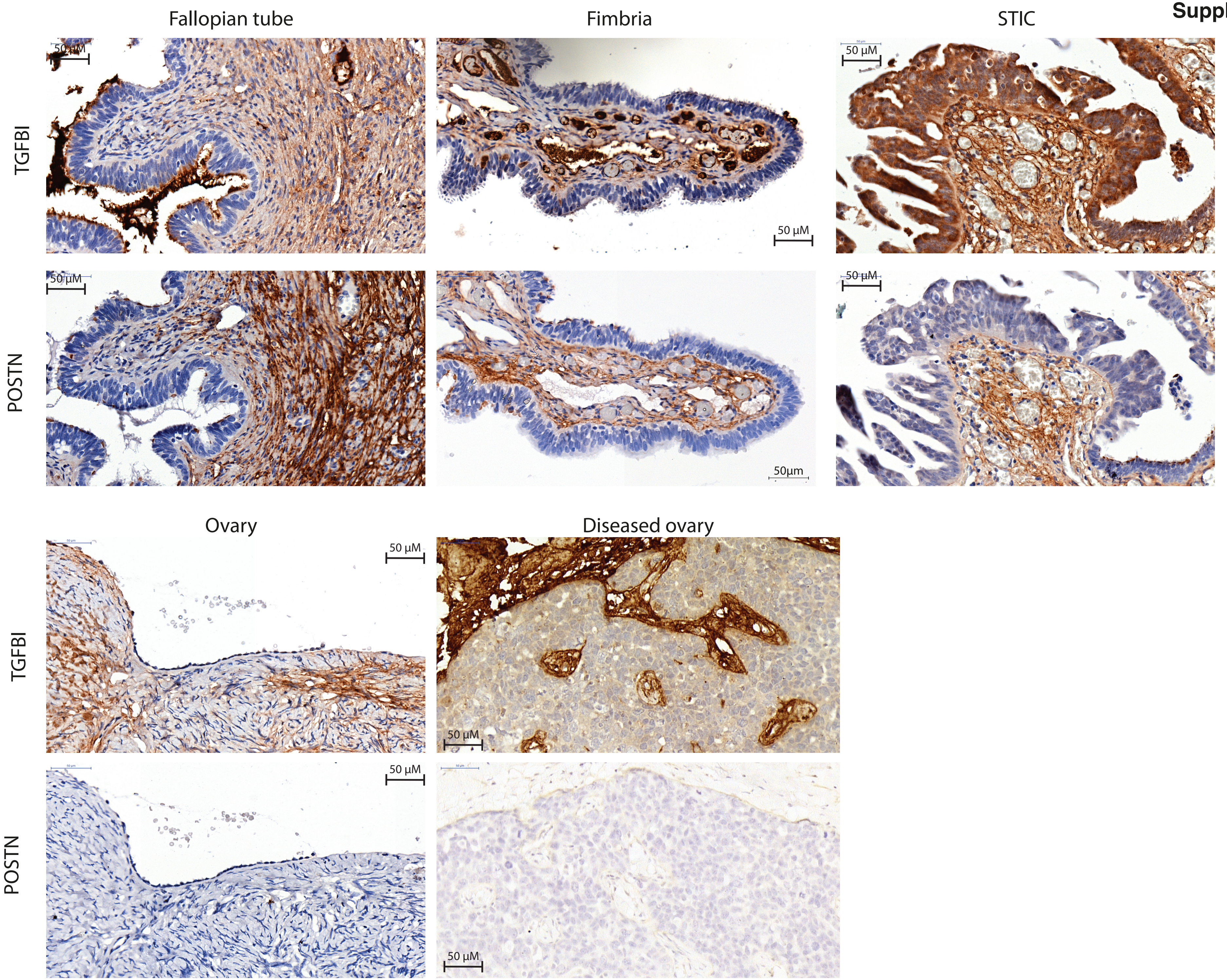

Supplementary figure 1: IHC of TGFBI and POSTN. Representative images shown in figure 1E in lager format, from fallopian tube (n=4), fimbria (n=4), STIC (n=7), ovary (n=6) and diseased ovary (n=3) tissues.

Supplementary figure 1: IHC of TGFBI and POSTN. Representative images shown in figure 1E in lager format, from fallopian tube (n=4), fimbria (n=4), STIC (n=7), ovary (n=6) and diseased ovary (n=3) tissues.
