# Supplementary material for: TGFBI Production by Macrophages Contributes to an Immunosuppressive Microenvironment in Ovarian Cancer
Source: Cancer Res. 2021 Sep 24;81(22):5706–19. doi: 10.1158/0008-5472.CAN-21-0536 (PMC9397609; doi:10.1158/0008-5472.CAN-21-0536)
Supplement: Figure S2 — Effect of macrophages and FTSE and HGSOC cell lines co-culture on CD163 and CD206 expression [file can-21-0536_figure_s2_suppsf2.pdf]

Supplementary Figure 2

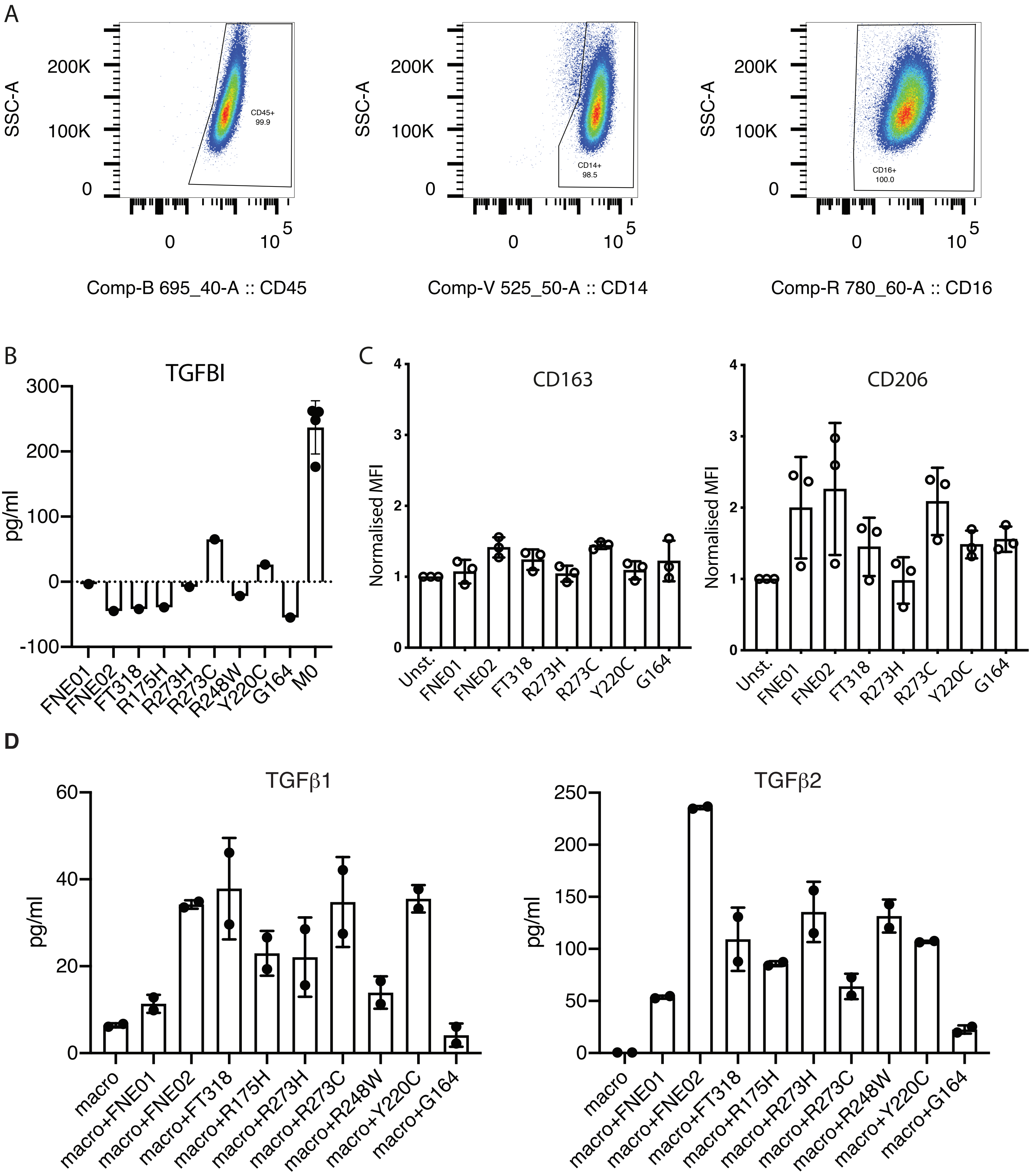

Supplementary figure 2: Effect of macrophages and FTSE and HGSOC cell lines co-culture on CD163 and CD206 expression. A Gating strategy for macrophages used in figures 3 and 4 in flow cytometry experiments. **B** TGFBI secretion of FTSE cell lines (wild type [FNE01, FNE02, FT318], n=1 and mutant p53 [R175H, R273H, R273C, R248W, Y220C], n=1) and a HGSOC cell line (G164) and M0 macrophages (n=4). **C** CD206 and CD163 expression post co-culture with FTSE and HGSOC cell lines. Data normalised to unstimulated macrophages, mean ± SD. Unstimulated macrophages (n=3) versus macrophages (three different PBMC donors) co-cultured with FNE01 (n=3), FNE02 (n=3), FT318 (n=3), R273H (n=3), R273C (n=3), Y220C (n=3), G164 (n=3). Statistical significance determined using one-way ANOVA (One-way analysis of variance; Dunnett's multiple comparisons test). ns= $p>0.05$. **D** TGFβ1 and TGFβ2 secretion of macrophages alone (macro) or co-cultured with FTSE cell lines (wild type [FNE01, FNE02, FT318] and mutant p53 [R175H, R273H, R273C, R248W, Y220C]) and a HGSOC cell line (G164). Supernatants from the experiment shown in Figure 4 E were tested by ELISA. Data are mean ± SD for two experiments.
