# Supplementary material for: TGFBI Production by Macrophages Contributes to an Immunosuppressive Microenvironment in Ovarian Cancer
Source: Cancer Res. 2021 Sep 24;81(22):5706–19. doi: 10.1158/0008-5472.CAN-21-0536 (PMC9397609; doi:10.1158/0008-5472.CAN-21-0536)
Supplement: Figure S3 — Effect of anti-TGFBI treatment in the HGS2 model [file can-21-0536_figure_s3_suppsf3.pdf]

Supplementary figure 3

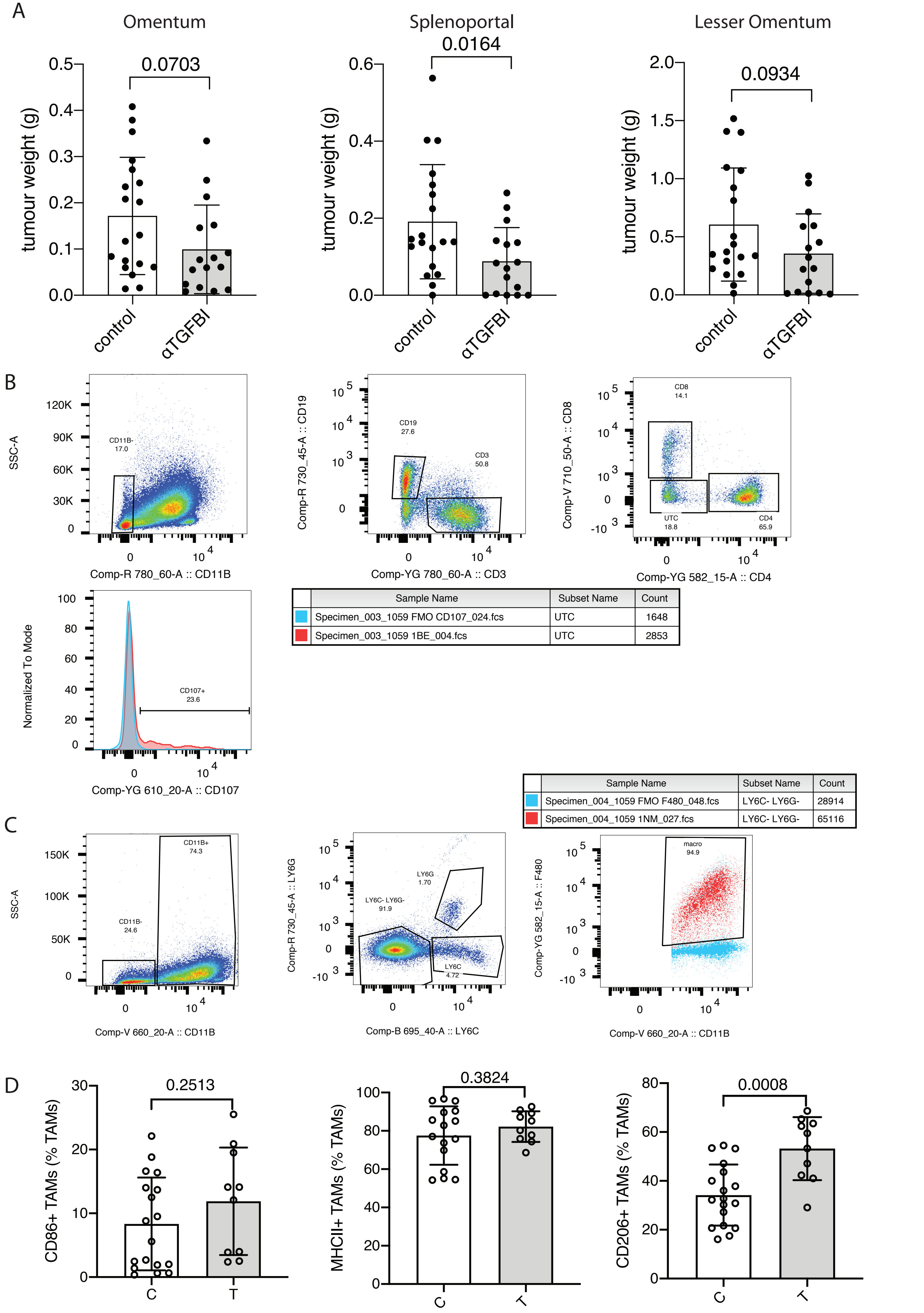

Supplementary figure 3: **Effect of anti-TGFBI treatment in the HGS2 model. A** Omentum , SP and LO tumor weight for mice injected with HGS2 and treated for three weeks with anti-TGFBI, starting at week 7 (n=18 for controls, n=10 for anti-TGFBI treated).**B, C** Gating strategy for the main populations analysed by flow cytometry from mouse tumors. Events were gated as single cells, CD45+, FVD (fixable viability dye)- and then as show for lymphocytes (B) and myeloid cells (C). CD107+ UTCs are overlayed to the CD107 FMO and F4/80+ Ly6C- Ly6G- are overlayed to the F4/80 FMO. **D** Percentage of macrophages positive for CD86, MHCII and CD206 in omental tumors from control-treated and anti-TGFBI treated mice (n=18 for controls, n=10 for anti-TGFBI treated). Statistical significance was determined using t-test.
